# Planktonic and sedimentary bacterial diversity of Lake Sayram in summer

**DOI:** 10.1002/mbo3.281

**Published:** 2015-08-03

**Authors:** Lei Fang, Lei Chen, Yuan Liu, Wei Tao, Zhongzhe Zhang, Haiying Liu, Yong Tang

**Affiliations:** 1 School of Marine Science and Environmental Engineering, Dalian Ocean UniversityDalian, 116023, China; 2 School of Life Sciences, Liaoning Normal UniversityDalian, 116029, China

**Keywords:** Lake Sayram, bacterial diversity, planktonic, sedimentary, 16S rRNA metagenomics, physicochemical parameters

## Abstract

Lake Sayram is an ancient cold water lake locating at a mountain basin in Xinjiang, China. The lake water is brackish, alkaline, unpolluted, and abundant in SO_4_^2−^ and Mg^2+^. The lacustrine ecosystem of Lake Sayram has been intensely investigated. However, profiles of the microbial communities in the lake remain largely unknown. In this study, taxonomic compositions of the planktonic and sedimentary bacterial communities in Lake Sayram were investigated using 16S rRNA metagenomics. The lacustrine bacterial communities were generally structured by environmental conditions, including the hydrological and physicochemical parameters. Proteobacteria was the dominating phylum. In the lake water, the genera *Acinetobacter* and *Ilumatobacter* held an absolute predominance, implying their metabolic significance. In the bottom sediment, biogeochemically significant bacteria and thermophilic or acidothermophilic extremophiles were recovered. In contrast to the planktonic bacteria, an appreciable portion of the sedimentary bacteria could not be classified into any known taxonomic unit, indicating the largely unknown bacteriosphere hiding in the bottom sediment of Lake Sayram.

## Introduction

Lake Sayram is a cold water lake locating at a mountain basin in Xinjiang, China, with an average altitude of 2071.9 m and an average annual atmospheric temperature of 0.5°C (Wang and Dou 1998). The lake water is mainly fed by rain water, snow water and underground water (Ma et al. 2003). It is sulfate-type brackish with total dissolved solids (rms) at around 3 g/L (Hao et al. 2013). Lake Sayram is a fault trough lake formed at the Himalayan orogeny 70 million years ago and its bottom sediments from the quaternary period record the entire history of the geomorphic development of West Tianshan Mountains as well as the shaping effect conferred by the fossil glacier (Wang et al. 2000). Aside from the rain alluvium at the shallow bottom of the west side, sands and rocks largely consist of the lake bottom and the sediments are composed of colloidal calcareous matter and quartzitic sands (Wei et al. 1994; Chen et al. 2014). Surrounded by high mountains, this closed lake is nearly free from direct influences of industrial and anthropogenic activities, resulting in an almost balanced lacustrine ecosystem (Yang et al. 2000). There used to be no fish living in Lake Sayram. In 1980s, some cold water fishes were introduced and from then on fishery industry has been developed (Guo et al. 2003).

A variety of investigations on the lacustrine ecosystem of Lake Sayram have been carried out. The lake is oligotrophic according to physicochemical parameters of the lake water (Guo et al. 2003). Investigations conducted by our group show that the typical aquatic plant is *Potamogeton pectinatus*; planktonic organisms include algae (Euglenophyta, Cyanophyta, Bacillariophyta, and Chlorophyta), protozoa (*Hemiophrys* sp. and *Tetrahymena* sp.), *Daphniopsis tibetana*, *Diatomid* sp. and rotifers; benthic fauna include *Gammrus* sp., *Chironomus* sp., etc. (Tang et al. unpubl. data).

However, profiles of the microbial communities in Lake Sayram remain largely unknown. In a lacustrine ecosystem, microorganisms play a pivotal role in driving the biogeochemical processes (Debroas et al. 2009). First, the photosynthetic and chemolithotrophic microorganisms are the main contributor of the primary productivity. Second, the chemoorganotrophic and chemoheterotrophic microorganisms are actively involved in the decomposition of organic matters. Third, appreciable amounts of microorganisms are the foodstuff for filter-feeding organisms and form the foundation of the aquatic food web (Wang et al. 2004). Therefore, it is of great interest to study the microbial communities in Lake Sayram.

In our study, the planktonic and sedimentary bacterial diversity of Lake Sayram in summer was investigated using 16S rRNA metagenomics. Physicochemical parameters of the lake water and sediments were examined as well. Correlation between the hydrological and physicochemical properties of the lake and the structure of the lacustrine bacterial communities were discussed.

## Materials and Methods

### Water samples collection

Conditions of each sampling site are listed in Table1. Water depths ranged from 38.5 m (at Site 3) to 89.0 m (at Site 4). Water samples were taken from Sites 1, 3, 4, 5, and 6. For Sites 1, 3, 5, and 6, lake water at the surface and the bottom layers was collected in equal volume and pooled to roughly represent the whole water column at individual site. Five liters of the surface water (1 m below the surface) and 5 L of the bottom water (1 m above the bottom) were collected and mixed (total volume was 10 L). For Site 4 which was located at the lake center, water from the surface, the bottom, and two middle layers was collected for mixing. Five liters of water at different depths (1 m, 20 m, 40 m below the surface, and 1 m above the bottom) were collected and mixed (total volume was 20 L). It should be noted that inclusion of the middle layered water samples would affect the structure of the bacterial communities investigated. Therefore, the planktonic bacterial diversity obtained here could be used for a horizontal comparison only. In total, five water samples were collected for subsequent planktonic microorganisms collection as well as physicochemical parameters assay.

**Table 1:** Conditions of the sampling sites in Lake Sayram

Sampling site	Date	Lat, Long	Sample type	Depth (m)	Transparency (m)	Layer	Temperature (°C)	Salinity (‰)	DO (mg/L)	pH
1	30/07/2014	44.384°N, 81.137°E	W, S	42.0	9	Sur	12.28	2.46	7.92	9.07
						Bot	4.93	2.60	9.69	9.08
2	30/07/2014	44.410°N, 81.081°E	S	60.0	6	Sur	13.69	2.48	9.02	9.10
						Mid	7.34	2.59	8.56	9.11
						Bot	4.92	2.63	10.04	9.11
3	30/07/2014	44.390°N, 81.039°E	W	38.5	6	Sur	13.80	2.44	7.52	9.13
						Bot	7.03	2.54	8.85	9.15
4	30/07/2014	44.359°N, 81.093°E	W, S	89.0	10	Sur	13.61	2.49	7.29	9.08
						Mid (20 m)	8.71	2.59	7.85	9.09
						Mid (40 m)	4.12	2.65	8.00	9.08
						Bot	3.22	2.65	9.75	9.08
5	31/07/2014	44.338°N, 81.063°E	W, S	63.0	4	Sur	11.56	2.51	7.71	9.11
						Bot	5.94	2.60	8.71	9.08
6	31/07/2014	44.333°N, 81.140°E	W, S	47.1	6	Sur	12.31	2.52	8.05	9.06
						Bot	4.18	2.66	8.81	9.05

Lat, latitude; Long, longitude; DO, dissolved oxygen; W, water; S, sediment; Sur, surface layer; Bot, bottom layer; Mid, middle layer.

To collect planktonic microorganisms, five liters of the mixed water at different sites was prefiltered using 1.6 *μ*m pore-size GF/A filter paper (Whatman®, UK) and then the filtrate was filtered through a 0.22 *μ*m pore-size filter membrane (Whatman®, NJ, USA). Microorganisms ranging from the size of 0.22 *μ*m to 1.6 *μ*m were thus collected onto the 0.22 *μ*m pore-size microfiltration membrane, which was transported on ice and stored at −20°C for subsequent DNA extraction.

To collect planktonic photosynthetic microorganisms, 5 L of the mixed water at various sites was filtered through a 0.45 *μ*m pore-size filter membrane. Organisms collected onto the 0.45 *μ*m pore-size filter membrane were subject to Chlorophyll a (Chl *a*) extraction and quantification.

### Sediment samples collection

Surface sediments were collected using a sediment sampler made of stainless steel (VanVeen Hydrobios series, Hydro-Bios, Kiel, GER). For sites 1, 4, 5, and 6, sediment samples were collected from the lake bottom in parallel with water sampling. However, at site 3, sediment collection was failed, which could be due to the bare rocked bottom. As an alternative, sediment was collected from site 2, which was on the north east of site 3. For DNA extraction, around 5 g of the sediments were transferred to a sterile plastic bag using an autoclaved stainless steel spoon, transported on ice and stored at −20°C. For physicochemical parameters assay, around 100 g of the sediments were transferred to a valve bag, transported at room temperature, and air dried for storage.

### Analysis of physicochemical parameters of water and sediment

Transparency was determined using the Disk method. For lake water samples, salinity, concentrations of DO (dissolved oxygen), temperature, and pH were measured in situ using a YSI 6600 multifunction water quality monitoring instrument (Fondriest Environmental, OH, USA). Chemical parameters were investigated following the Chinese National Standards – the Environmental Quality Standards for Surface Water (GHZBI-121999). COD (chemical oxygen demand) was measured using the potassium dichromate oxidation method. The concentration of ammonia was determined using the Nessler's reagent method. The concentration of nitrate was analyzed using the cadmium column reduction method. The concentration of nitrite was examined using the diazo and azo colorimetry method. The content of dissolved inorganic phosphorus (DIP) was determined using the phosphorus molybdenum blue colorimetric method. The concentration of silicate was measured using the silicon molybdenum blue colorimetric method. The concentrations of bicarbonateion and carbanion were determined using the acid titration method. The concentration of chloride ion was measured using the argentometric method. The concentrations of sulfate ion, calcium ion, and magnesium ion were examined using the EDTA (Ethylenediaminetetraacetic acid) titration method. The sediment samples were first acidated using hydrochloric acid and then subject to total organic carbon (TOC) examination using vario PYRO cube element analyzer (Elementar®, Hanau, GER). The contents of heavy metal elements in the sediments were examined using PE AAnalyst Model 300 atomic absorption spectrophotometer (PerkinElmer®, Waltham, MA, USA).

### Determination of the Chl *a* content

To determine the Chl *a* content, cells collected onto the 0.45 *μ*m pore-size filter membrane were resuspended with 80% acetone and incubated at 4°C for overnight. Cell debris was cleared by centrifugation. The resulting supernatant was subjected to OD measurement at the wavelength of 645 and 663 nm, with the 80% acetone as the blank. Total Chl *a* content was estimated using the formula Chl *a* (mg/L) = 12.72OD_663_ − 2.59OD_645_ and then converted to *μ*g/L.

### DNA extraction, PCR amplicon libraries construction and sequencing

Total DNA from the water samples was extracted using the E.Z.N.A.® Water DNA Kit (OMEGA bio-tek, Norcross, GA, USA) and total DNA from the sediment samples was isolated using the E.Z.N.A.® Soil DNA Kit (OMEGA bio-tek, Norcross, GA, USA), following the manufacturer's instructions. Integrity and concentration of each environmental DNA were investigated using gel electrophoresis.

The quality-ensured DNA was quantified using the Qubit® 2.0 DNA assay kit (Life Technologies, Waltham, MA, USA) and used as the template to amplify the V3 and V4 regions of bacterial 16S rRNA gene. The universal adaptors for the 16S rRNA V3 to V4 region on Illumina MiSeq® System (Illumina, San Diego, CA, USA) were fused to the 5′ end of primer 341F, 5′-CCTACACGACGCTCTTCCGATCTN-CCTACGGGNGGCWGCAG-3′, and primer 805R, 5′-GACTGGAGTTCCTTGGCACCCGAGAATTCCA-GACTACHVGGGTATCTAATCC (the adaptors sequences were underlined). The 50 *μ*L of PCR mix contained 10 ng of genomic DNA, 2.5 units of Platinum Taq polymerase, 1 × PCR buffer, 100 *μ*mol/L of dNTP mix, and 0.5 *μ*mol/L of each primer. Cycling conditions were an initial denaturation at 94°C for 3 min; five cycles of 94°C for 30 sec, 45°C for 20 sec, and 65°C for 30 sec; 20 cycles of 94°C for 20 sec, 55°C for 20 sec, and 72°C for 30 sec; and a final extension at 72°C for 5 min. The PCR products were gel electrophoresed and the sharp bands with the correct size were excised and purified using the gel purification kit (Sangon, Shanghai, China). The purified PCR products were quantified using the Qubit® 2.0 DNA assay kit (Life Technologies, Waltham, MA, USA). The same amount of PCR products from each environmental sample was pooled and subject to reversible-terminator sequencing by synthesis technology using Illumina MiSeq® System (Illumina, San Diego, CA, USA). For each sample, no less than 10,000 clean reads were recovered.

### Bioinformatic analyses

Pair-end sequences were first merged using the FLASH software (http://sourceforge.net/projects/flashpage/, Version 1.2.3 ) and then quality controlled using the Prinseq software (http://prinseq.sourceforge.net/, Version 0.20.4). Maximum mismatch ratio for the overlapping regions was set at 0.1 and minimum length for the merged sequences was set at 50 bp. Those merged sequences with low complexity were also filtered. Those qualified sequences were then attributed to individual environmental samples according to specific barcodes. The nontarget regions were trimmed. The sequencing errors were corrected using pre.cluster of the mother software and the chimeric sequences were removed using chimeras.uchime. For individual samples, total sequences were clustered into different OTUs (operational taxonomic units) based on sequence similarities (set as 0.97) using uclust. For each OTU, representative sequences were phylogenetically analyzed to various levels (domain, phylum, class, order, family, and genus) using the Bayesian algorithm of RDP (ribosomal database project) classifier. Relative abundance of each taxonomic unit was also calculated using the Naïve Bayesian assignment of RDP classifier.

## Results

### Locations of the sampling sites

**Figure 1:**
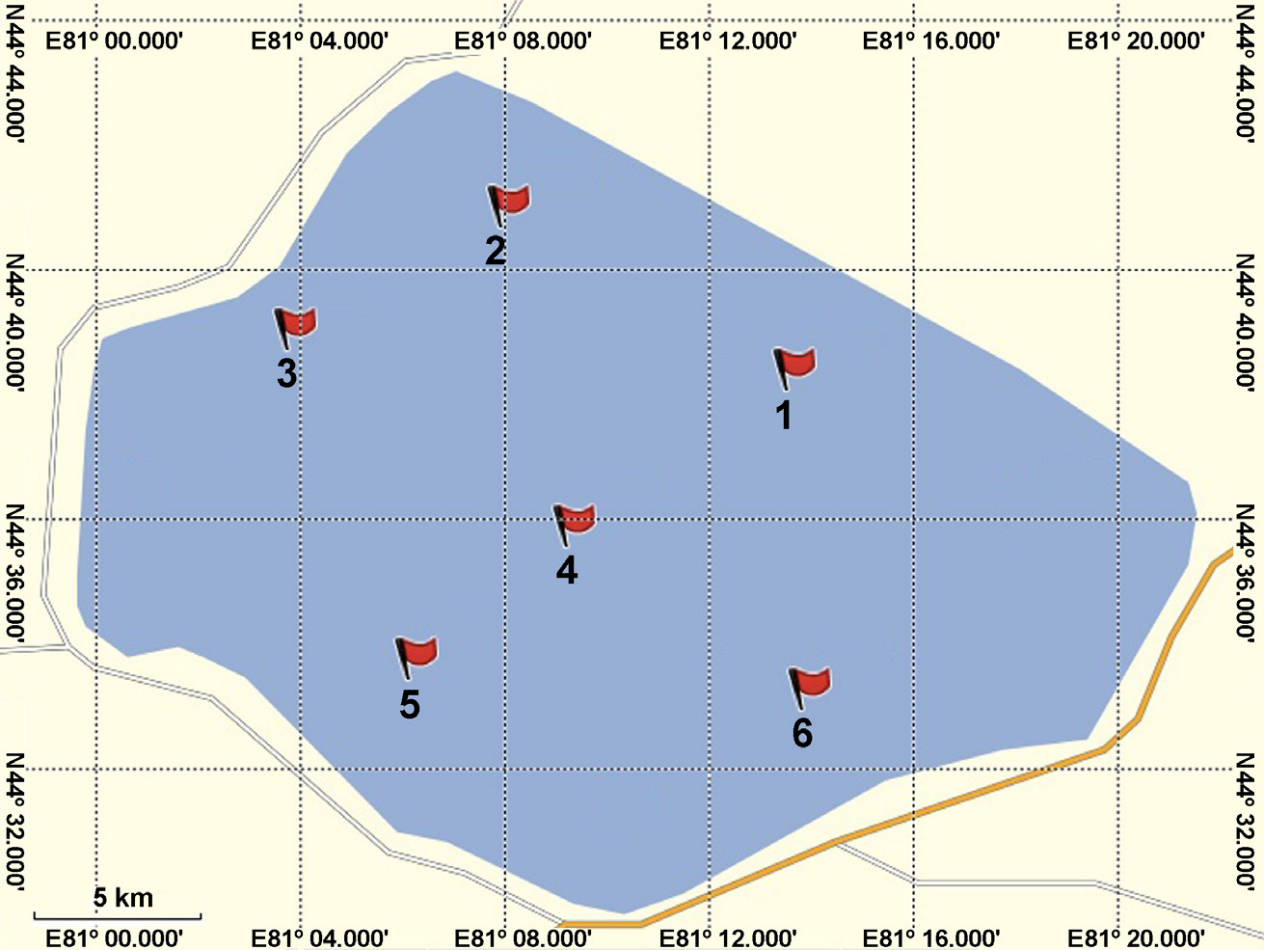
Locations of sampling sites in Lake Sayram. For Sites 1, 4, 5, and 6, both water and sediments were sampled. For Site 3, only water sample was taken. For Site 2, only sediment sample was collected.

Towards a comprehensive investigation of the bacterial diversity in Lake Sayram, six independent sites were selected for water and sediment sampling in July 2014 (Fig.1). Sites 1, 2, 3, and 4 were not easily accessed by human beings. Site 1 was located in the northeast of the lake, which was near the influx of spring water. Sites 2 and 3 were in the north and west of the lake, respectively, both of which were close to the input of seasonal rivers. Site 4 was at the centre of the lake. In contrast, Sites 5 and 6 were more prone to the influences of human activities. Site 5 was within a short distance from the expressway, which was south to the lake. Site 6 was near the pasture of the east lakeshore, where tourism was burgeoning.

### Physicochemical parameters of the lake water

Physicochemical parameters of the lake water examined on site are listed in Table1. The lake water was brackish, alkaline, cold, oxidized, and clear. The average salinity was around 2.56‰ and the average pH was around 9.1. Temperatures of the lake water decreased with the increasing water depths. Water temperatures ranged from 11.56 °C to 13.80 °C at the surface layer and varied from 3.22 °C to 7.03 °C at the bottom layer. The lake water was abundant in DO and the concentrations of DO ([DO]) were negatively correlated with water temperatures. [DO] at the surface layer was between 7.29 and 8.05 mg/L, whereas [DO] at the bottom layer was between 8.71 and 10.04 mg/L. Transparency of the lake water was 10 m at Site 4 (the lake center), 9 m at Site 1, 6 m at Sites 2, 3, and 6, and 4 m at Site 5, respectively. The lowest transparency was detected at the site adjacent to the express way.

Other chemical parameters (COD, nutrient salts, Chl *a*, anions, cations) of the lake water are shown in Table2. The lake water was clean, NO_3_^−^ type, phosphorus-limited and abundant in SO4^2−^, Cl^−^, and Mg^2+^. The average COD_Cr_ (COD) was lower than 1 mg/L. NO_3_^−^-N was the major type of inorganic nitrogen, followed by NH_4_^+^-N and NO_2_^−^-N. The distribution of dissolved NO_3_^−^-N was nonuniform in this lake. The highest concentration was 0.44 ± 0.03 mg/L at Site 1, which was twofold of the lowest concentration detected at Site 5 (0.21 ± 0.04 mg/L). Concentrations of NH_4_^+^-N ranged from 30.47 ± 3.02 *μ*g/L (Site 6) to 19.70 ± 0.48 *μ*g/L (Site 5). Total DIP was found to be most abundant at Site 5 (29.26 ± 1.85 *μ*g/L) and most scarce at Site 3 (3.70 ± 0.28 *μ*g/L). In terms of nitrogen to phosphorus ratio (N/P), the highest value was 127.9 at Site 1 and the lowest 8.0 at Site 5. Phosphorus was therefore the limiting factor of the primary productivity in the lake water. The contents of Chl *a* were examined to indicate the biomass of photosynthetic microorganisms. In general, concentrations of Chl *a* were positively correlated with the transparency. The highest content of Chl *a* was detected at Site 1 (0.76 ± 0.05 *μ*g/L), and the lowest was detected at Site 5 (0.44 ± 0.04 *μ*g/L). In another word, the clearer the lake water was, the more light could penetrate and the more photosynthetic microorganisms could grow. Of the four types of anions (Cl^−^, SO_4_^2−^, HCO_3_^−^, and CO_3_^2−^) examined, Cl^−^ and SO_4_^2−^ were the two dominant types and shared comparable concentrations. Mg^2+^ was an abundant cation in the lake water, with a concentration ranging from 0.36 to 0.38 g/L across all the sampling sites.

**Table 2:** Chemical parameters of the lake water

Site	COD (mg/L)	NH_4_^+^-N (*μ*g/L)	NO_3_^−^-N (mg/L)	NO_2_^−^-N (*μ*g/L)	DIN (mg/L)	DIP (*μ*g/L)	N/P	Silicate (*μ*g/L)	Cl^−^ (g/L)	SO_4_^2−^ (g/L)	HCO_3_^−^ (g/L)	CO_3_^2−^ (g/L)	Ca^2+^ (mg/L)	Mg^2+^ (g/L)	Chl *a* (*μ*g/L)
1	0.96 ± 0.02	29.93 ± 1.31	0.44 ± 0.03	3.03 ± 0.04	0.47	3.70 ± 0.28	127.9	68.47 ± 1.80	0.84 ± 0.06	0.84 ± 0.02	0.60 ± 0.01	0.11 ± 0.00	6.41 ± 0.25	0.38 ± 0.00	0.76 ± 0.05
3	1.02 ± 0.08	21.92 ± 3.80	0.37 ± 0.01	2.75 ± 0.04	0.39	9.60 ± 0.74	40.9	74.21 ± 5.16	0.86 ± 0.01	0.88 ± 0.03	0.53 ± 0.03	0.11 ± 0.03	13.63 ± 0.56	0.36 ± 0.00	0.54 ± 0.02
4	0.66 ± 0.05	21.90 ± 1.22	0.28 ± 0.01	3.10 ± 0.03	0.30	20.74 ± 0.49	14.6	83.64 ± 6.17	0.85 ± 0.03	0.88 ± 0.05	0.63 ± 0.01	0.11 ± 0.01	11.62 ± 1.02	0.36 ± 0.01	0.60 ± 0.06
5	0.84 ± 0.11	19.70 ± 0.48	0.21 ± 0.04	3.31 ± 0.04	0.23	29.26 ± 1.85	8.0	87.74 ± 2.32	0.83 ± 0.01	0.88 ± 0.05	0.61 ± 0.01	0.10 ± 0.01	10.42 ± 0.90	0.36 ± 0.01	0.44 ± 0.04
6	0.68 ± 0.04	30.47 ± 3.02	0.28 ± 0.02	3.03 ± 0.04	0.32	15.50 ± 0.46	20.6	37.72 ± 1.16	0.83 ± 0.04	0.91 ± 0.04	0.58 ± 0.06	0.11 ± 0.02	7.21 ± 0.13	0.37 ± 0.00	0.50 ± 0.03

Values shown were the average ± standard deviation from three individual replicates. COD, chemical oxygen demand; DIN, dissolved inorganic nitrogen; DIP, dissolved inorganic phosphorus; N/P, nitrogen to phosphorus ratio; Chl *a*, chlorophyll a.

To sum up, the water in Lake Sayram was unpolluted and most of the physicochemical parameters of the lake water met the requirements of Grade 1 of the Chinese National Standards – the Environmental Quality Grading Standards for Surface Water (GB 3838 – 88). Content of sulfate and the total hardness conferred by Mg^2+^ and Ca^2+^ met the requirements of Grade 3.

### Chemical parameters of the lake bottom sediments

**Figure 2:**
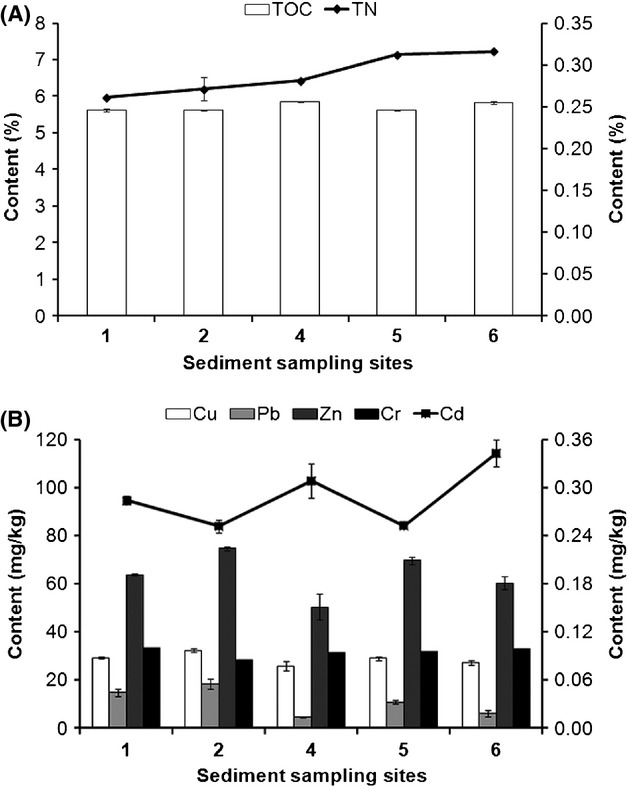
Chemical parameters of the bottom sediments in Lake Sayram. (A) Contents of total organic carbon (TOC, the bar chart) and total nitrogen (TN, the line chart) in the sediment samples. (B) Contents of Cu, Pb, Zn, Cr (the bar charts) and Cd (the line chart) in the sediment samples.

For the sediment samples collected from Lake Sayram, chemical parameters including contents of TOC, total nitrogen (TN) and heavy metals (Cu, Pb, Zn, Cr, and Cd) were also investigated, as shown in Figure2 and Table3. Here, TOC was used to reflect the content of organic matter in sediments (Li 2011). The contents of TOC across different sampling sites ranged from 5.60% to 5.90%, and the contents of TN varied from 0.26% to 0.32%. In general the metal elements Cu, Cr, and Cd were evenly distributed in the lake bottom. Pb and Zn shared a similar distribution pattern across all the sampling sites, with the lowest abundance found at Site 4 and the highest abundance detected at Site 2. In terms of contents, none of the five typical heavy metals exceeded the Environmental Quality Standards for Soils (GB 15618 – 1995), indicating that the bottom of Lake Sayram remained undisturbed from human activities.

**Table 3:** Chemical parameters of the lake bottom sediments

Site	TOC (%)	TN (%)	Cu (mg/kg)	Pb (mg/kg)	Zn (mg/kg)	Cr (mg/kg)	Cd (mg/kg)
1	5.63 ± 0.04	0.26 ± 0.00	29.21 ± 0.45	14.82 ± 1.56	63.76 ± 0.40	33.34 ± 0.53	0.28 ± 0.01
2	5.62 ± 0.00	0.27 ± 0.01	32.32 ± 0.68	18.43 ± 2.05	74.71 ± 0.76	28.43 ± 3.47	0.25 ± 0.01
4	5.85 ± 0.00	0.28 ± 0.00	25.69 ± 1.93	4.57 ± 0.18	50.30 ± 5.28	31.27 ± 2.67	0.31 ± 0.02
5	5.62 ± 0.02	0.31 ± 0.00	29.01 ± 0.78	10.89 ± 0.73	69.73 ± 1.51	31.89 ± 2.88	0.25 ± 0.00
6	5.83 ± 0.04	0.32 ± 0.00	27.36 ± 0.98	6.14 ± 1.45	60.24 ± 2.72	33.13 ± 1.11	0.34 ± 0.02

Values shown were the average ± standard deviation from three individual replicates. TOC, total organic carbon; TN, total nitrogen.

### Planktonic and sedimentary bacterial community compositions in Lake Sayram

Metagenomics based on the 16S rRNA gene was performed to investigate the structure of microbial communities in the lake water and the bottom sediments. For the water samples, 14 phyla, 23 classes, 35 orders, 69 families, and 134 genera of bacteria (or prokaryotes) were recovered, whereas for the sediment samples, 25 phyla, 57 classes, 71 orders, 158 families, and 413 genera of bacteria were retrieved (Table4).

**Table 4:** Number of bacterial taxonomic units recovered from the water and sediment samples

Sample type	Sampling site	Number of taxonomic units				
		Phylum	Class	Order	Family	Genus
Water	1	11	18	28	52	80
	3	9	14	22	43	73
	4	9	16	24	42	77
	5	12	20	22	54	86
	6	11	18	27	47	76
	Total	14	23	35	69	134
Sediment	1	24	47	62	107	232
	2	24	46	62	117	250
	4	24	48	57	100	206
	5	25	55	66	126	286
	6	24	51	62	105	219
	Total	25	57	71	158	413

**Figure 3:**
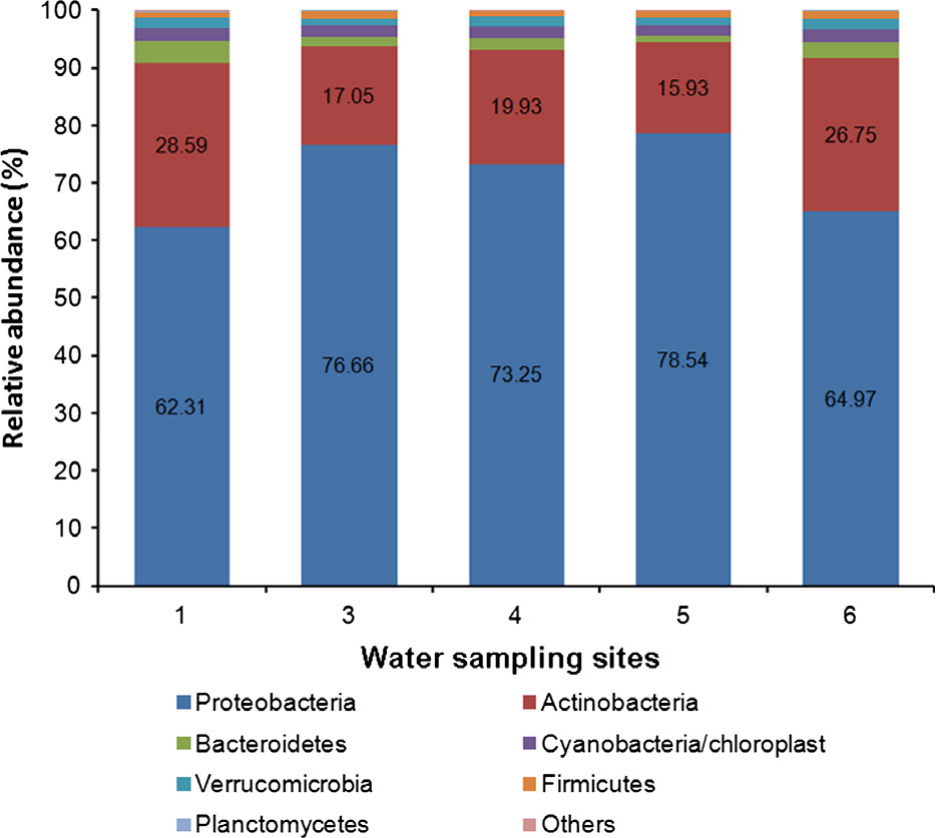
The planktonic bacterial diversity of Lake Sayram at the phylum level. “Others” includes Gemmatimonadetes, Chlorobi, Chloroflexi, Acidobacteria, WS3, Armatimonadetes, SR1, and unidentified.

The taxonomic composition at the phylum level of the planktonic bacterial communities in Lake Sayram is shown in Figure3 and Table S1. Proteobacteria held the overwhelming predominance and its relative abundance was above 60%. The second predominant phylum was Actinobacteria, which constituted 15.93–28.59% of the bacterial communities. The additive abundance of the two most dominant phyla, was above 90% across all the sampling sites. Bacteroidetes, Cyanobacteria/Chloroplast, Verrucomicrobia, Firmicutes, and Planctomycetes were the other five phyla which were universally detected. Another seven phyla were recovered from one or several sampling sites and their relative abundance was below 0.02%.

Figure S1 and Table S2 showed the taxonomic composition of the planktonic bacterial communities at the genus level. *Actinobacter* was the most predominant genus, with relative abundance ranging from 53.23% to 70.02%. *Ilumatobacter* was the second largest genus, constituting 10–20% of the planktonic bacterial communities. Other dominant genera with appreciable relative abundance (average values above 0.1%) in the planktonic bacterial communities included *Loktanella*, *GPIIa*, *Cryobacterium*, *Agrococcus*, *Ornithinibacter*, *Spartobacteria_genera_incertae_sedis*, *Pseudomonas*, *Algoriphagus*, *Psychrobacter*, *Limnohabitans*, *Planococcus*, *Porphyrobacter*, *Rheinheimera*, *Luteolibacter*, *Brumimicrobium*, *Methylophilus*, *Pasteuria*, *Kerstersia*, *Gracilimonas*, *Blastopirellula*, and *Exiguobacterium*. For the individual samples, percentages of these genera were either varied or consistent.

**Figure 4:**
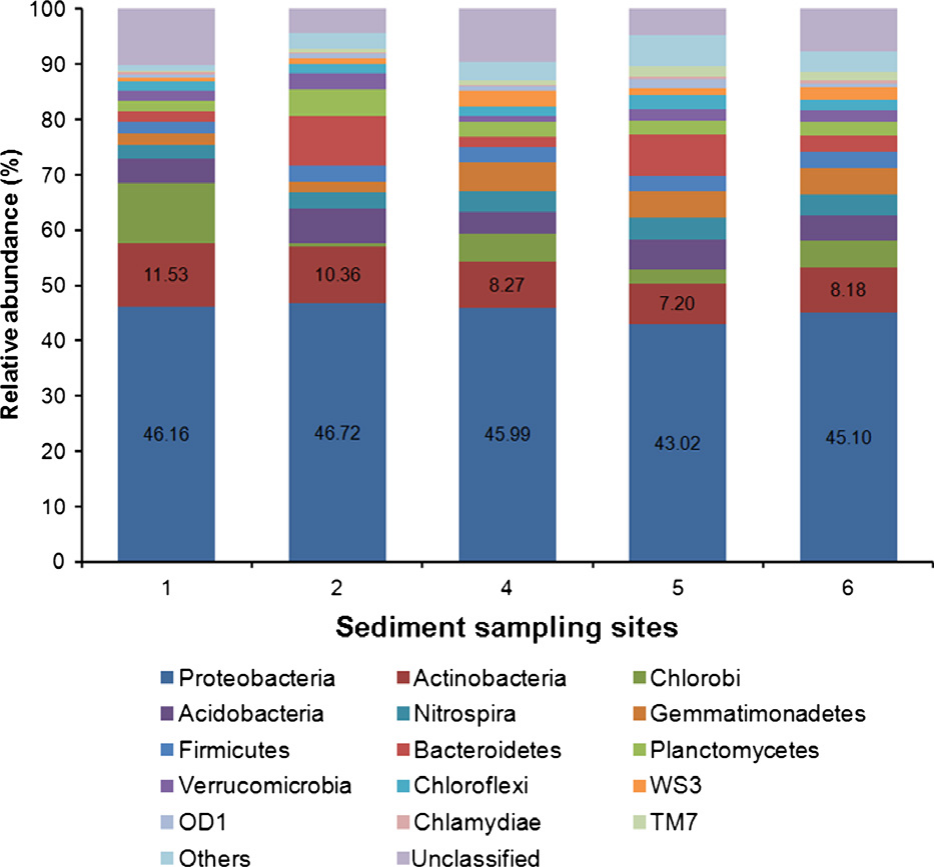
Sedimental bacterial diversity of Lake Sayram at the phylum level. “Others” includes Deinococcus-Thermus, Fibrobacteres, Elusimicrobia, Armatimonadetes, Cyanobacteria/Chloroplast, Spirochetes, Euryarchaeota, BRC1, SR1, and Thermodesulfobacteria.

The taxonomic composition at the phylum level of the sedimental bacterial communities in Lake Sayram is shown in Figure4 and Table S3. The phyla universally detected at all sediment samples included Proteobacteria, Actinobacteria, Acidobacteria, Chlorobi, Bacteroidetes, Gemmatimonadetes, Nitrospira, Planctomycetes, Firmicutes, Verrucomicrobia, Chloroflexi, etc. Their individual abundance was either consistent or varying across all the sedimental samples investigated. Proteobacteria was the largest phylum dominating the sedimental bacterial communities, with relative abundance ranging from 43.02% to 46.72%. Actinobacteria was in general the second most abundant phylum in the bacterial communities examined, with percentages varying from 7.20% to 11.53%. However, its predominance was not that absolute and within the same community some other phyla might hold an almost equal abundance. For example, at Site 1, Chlorobi was nearly as abundant as Actinobacteria and at Site 5, the relative abundance of Bacteroidetes was comparable to that of Actinobacteria.

Figure S2 and Table S4 show the taxonomic composition of the sedimental bacterial communities at the genus level. Dominant genera included *Ignavibacterium*, *Thioprofundum*, *Gemmatimonas*, *Thiobacter*, *Nitrospira*, *Acinetobacter*, *Ohtaekwangia*, *Gp16*, *Desulfohalobium*, *WS3_genera_incertae_sedis*, *Blastopirellula*, *Iamia*, *Methyloversatilis*, *TM7_genera_incertae_sedis*, *Ilumatobacter*, *Subdivision3_genera_incertae_sedis*, *Steroidobacter*, *OD1_genera_incertae_sedis*, *Aciditerrimonas*, etc. *Acinetobacter*, *Blastopirellula,* and *Ilumatobacter* dominated both the sedimentary and the planktonic bacterial communities. For each dominant genus, its distribution among the five sampling sites was highly diversified. In another word, relative abundance of the individual genus might differ a lot at different sites.

## Discussion

For both planktonic and sedimentary bacterial communities, the phylogenetic diversity of the minority bacteria (the rare bacterial biosphere) was much higher than that of the majority bacteria. For example, more than five phyla consisted of less than 10% of the planktonic bacterial community, whereas two phyla composed the rest over 90% portions. It was consistent with the famous statement that “everything is everywhere, but the environment selects” in environmental microbiology (De Wit and Bouvier 2006). The highly diverse rare biosphere might be of ecological significance in the evolution of Lake Sayram (Pedrόs-Aliό 2012).

The planktonic and sedimentary bacterial communities of Lake Sayram showed distinct structures, except that both of them were dominated by the phylum Proteobacteria. In comparing with the planktonic bacterial community, the sedimental counterpart displayed a much higher phylogenetic diversity, which might be due to the much more complicated physicochemical properties of sediments.

In addition, the planktonic bacteria were commonly found elsewhere in the environment, whereas an appreciable part of the sedimental bacteria remained unidentified and therefore might be environmentally unique. Nearly all the planktonic bacteria could be classified into various phyla within the Bacteria domain and about 90% of them could be grouped into different genera (Tables S1, S2). Those identified genera recovered from the lake water samples were widespread in the environment. However, 5.92–10.33% of the 16S rRNA clean reads recovered from the sedimental samples could not be classified into any phylum under the Bacteria domain. Furthermore, around 40% of the sedimentary reads could not be grouped into any known genus, revealing the largely unknown bacterial biosphere hiding in the bottom sediments of this ancient lake. Although Lake Sayram was a closed lake locating at a mountain basin, its lake water kept active material exchanging and energy transformation with the environment through atmospheric circulation, rain water precipitation, etc. Consequently, the lake water became an open system and its bacterial communities were determined by the environment. In contrast, the bottom sediments could not freely communicate with the environment and therefore were separated as exclusive niches to preserve the unique properties (including microorganisms) of this ancient lake. Bacteria inhabiting in the sediments of Lake Sayram, especially those unidentified, might be a reserve of ancient bacteria which were of geological value.

The genera *Actinobacter* and *Ilumatobacter* accounted for around 70% of the planktonic bacteria. As the taxa occupying bigger proportion in bacterial communities are correlated to the metabolic properties (Debroas et al. 2009), *Actinobacter* and *Ilumatobacter* might play a vital role in the metabolism of the lake water. Species of the *Actinobacter* genus were widely distributed in the environment, including soil, water, and sewage (Ventura et al. 2007; Servin et al. 2008; Ghai et al. 2011). Members of this genus were involved in the mineralization of organic matters in soil (Kästner et al. 1994). Type species of *Ilumatobacter* inhabited in marine environment such as sediment of the estuaries, sand of the coastal beach and marine sponges (Matsumoto et al. 2009; Khan et al. 2012).

The planktonic bacterial communities were largely structured by the physicochemical parameters of the lake water and inoculation of microbes from the water supply, which was in agreement with previous findings in other lakes (Humayoun et al. 2003; Jiang et al. 2006; Crump et al. 2012; Santofimia et al. 2013; Pjevac et al. 2015). In general, the planktonic bacteria were aerobic or facultatively aerobic/anaerobic, psychrophilic or psychrotolerant, and halotolerant. Many genera of psychrophilic/psychrotolerant bacteria recovered from the water samples were reported to inhabit in polar regions, which could be due to the input of snow water and cold spring water. These bacteria included *Loktanella* (Van Trappen et al. 2004), *Cryobacterium* (Zhang et al. 2007; Bajerski et al. 2011), *Algoriphagus* (Bowman et al. 2003), *Psychrobacter* (Bowman et al. 1997), *Planococcus* (Reddy et al. 2002), *Luteolibacter* (Jiang et al. 2012), *Brumimicrobium* (Bowman et al. 1997), etc. Among the five sampling sites, the genera *Cryobacterium* and *Agrococcus* were found to be most abundant at Site 1, which was closest to the entrance of cold spring water. Unlike the cold environment origin of *Crybacterium*, members of *Agrococcus* were widespread in the environment, including the frozen soil, sandstone surface, air, glacier, seaweed, etc. (Groth et al. 1996; Lee 2008).

The lake water also accommodated bacteria with considerable adaptability. For instance, the genera *Ornithinibacter* and *Exiguobacterium* were characterized by their ability to thrive in a wide range of temperature and pH (Frühling et al. 2002; Yumoto et al. 2004; Kim et al. 2005; Chaturvedi and Shivaji 2006; Crapart et al. 2007; Xiao et al. 2011; Raichand et al. 2012). Some species of *Exiguobacterium* could tolerate high levels of UV radiation and heavy metal stress.

*GPIIa*, *Porphyrobacter*, and *Methylophilus* appeared to contribute to the prokaryotic primary productivity of the lake water. *GPIIa* was the only genus that constituted the photosynthetic phylum Cyanobacteria/Chloroplast in the planktonic bacterial communities. Another possible photoautotrophic genus universally detected in the lake water samples was *Porphyrobacter*. Species of this genus inhabited in aquatic environments, such as fresh water, sea water, and hot spring (Fuerst et al. 1993; Rainey et al. 2003; Yoon et al. 2006; Furuhata et al. 2013). Some members of *Porphyrobacter* contained bacteriochlorophyll and were therefore photosynthetic (Fuerst et al. 1993; Hiraishi et al. 2002). Some species of *Porphyrobacter* were able to degrade biphenyl and dibenzofuran (Hiraishi et al. 2002). Another potential contributor to the planktonic primary productivity was the genus *Methylophilus*, a restricted facultative methanol utilizer (Jenkins et al. 1987). In addition to single carbon compounds, *Methylophilus* could also use a limited range of more complex organic compounds as the carbon and energy source (Jenkins et al. 1987).

The predominant sedimentary genera were widespread in the environment, distributing in multiple terrestrial and aquatic habitats. Majority of them were either anaerobic or facultatively anaerobic. The genera *Thioprofundum*, *Ohtaekwangia*, *Iamia,* and *Ilumatobacter* were of marine origin (Kurahashi et al. 2009; Matsumoto et al. 2009; Mori et al. 2011; Yoon et al. 2011; Roeselers and Newton 2012). The genus *Desulfohalobium* inhabited in hypersaline lakes (Ollivier et al. 1991; Jakobsen et al. 2006).

It was noteworthy that *Thioprofundum*, *Thiobacter,* and *Aciditerrimonas*, the predominant bacterial genera universally detected in the sediments of this alkaline cold water lake, were thermophilic or thermoacidophilic extremophiles (Hirayama et al. 2005; Itoh et al. 2011; Mori et al. 2011), implying that there might be thermal springs at the lake bottom. Similar with what was found elsewhere (i.e., the sea floor), thermal springs might also be the source of sulfur (S, H_2_S), methane (CH_4_), nitrogen (N_2_), and metals (Cu, Zn, Mn, Co, etc.), the elements or compounds constituting the lake bottom sediments.

Active players in biogeochemical processes (the sulfur cycle, the nitrogen cycle, and the carbon cycle) were retrieved from the sediment samples. Bacteria with antagonistic metabolic functions were found to co-predominate in the lake bottom sediments, indicating the way how an ecosystem acquired a metabolic balance. *Thioprofundum* and *Thiobacter* were obligately chemolithoautotrophic and thiosulfate-/sulfur-oxidizing, whereas *Desulfohalobium* was chemoorganotrophic and sulfate-reducing (Ollivier et al. 1991; Grice et al. 1998; Hirayama et al. 2005; Jakobsen et al. 2006; Mori et al. 2011). *Nitrospira* was nitrite-oxidizing, whereas *Steroidobacter* was nitrate-reducing. Similar with the *Methylophilus* genus detected in lake water, *Methyloversatilis*, another facultatively methylotroph, was recovered from the sediments (Kalyuzhnaya et al. 2006).

## Conclusion

This is the first comprehensive report on the planktonic and sedimentary bacterial diversity of Lake Sayram. Proteobacteria is the phylum dominating the lacustrine bacterial communities. Profiles of the bacterial communities in this ancient cold water lake are related to its hydrological and physicochemical properties. The planktonic bacteria are commonly found in the environment, whereas a considerable part of the sedimentary bacteria are unique and remain unidentified. In the planktonic bacterial community, psychrophilic/psychrotolerant and halotolerant genera of bacteria are frequently found. The genera *Acinetobacter* and *Ilumatobacter* hold an absolute predominance. In the sedimentary bacterial communities, biogeochemically significant bacteria and thermophilic or acidothermophilic extremophiles are detected.

## Supporting Information

**Table S1.** Taxonomic composition of the planktonic bacterial communities in Lake Sayram (at the phylum level). **Table S2.** Taxonomic composition of the planktonic bacterial communities in Lake Sayrama (at the genus level). **Table S3.** Taxonomic composition of the sedimental bacterial communities in Lake Sayram (at the phylum level). **Table S4.** Taxonomic composition of the sedimental bacterial communities in Lake Sayrama (at the genus level). **Figure S1.** The planktonic bacterial diversity of Lake Sayram at the genus level. **Figure S2.** Sedimental bacterial diversity of Lake Sayram at the genus level.
