# Supplementary material for: Planktonic and sedimentary bacterial diversity of Lake Sayram in summer
Source: Microbiologyopen. 2015 Aug 3;4(5):814–25. doi: 10.1002/mbo3.281 (PMC4618613; doi:10.1002/mbo3.281)
Supplement: Supplementary file 1 — Table S1. Taxonomic composition of the planktonic bacterial communities in Lake Sayram (at the phylum level). Table S2. Taxonomic composition of the planktonic bacterial communities in Lake Sayrama (at the genus level). Table S3. Taxonomic composition of the sedimental bacterial communities in Lake Sayram (at the phylum level). Table S4. Taxonomic composition of the sedimental bacterial communities in Lake Sayrama (at the genus level). Figure S1. The planktonic bacterial diversity of Lake Sayram at the genus level. Figure S2. Sedimental bacterial diversity of Lake Sayram at the genus level. [file mbo30004-0814-sd1.docx]

**Table S1.** Taxonomic composition of the planktonic bacterial communities in Lake Sayram (at the phylum level)

| Phylum | Relative Abundance (%) | | | | | |
| --- | --- | --- | --- | --- | --- | --- |
|  | Site 1 | Site 3 | Site 4 | Site 5 | Site 6 | Average |
| Proteobacteria | 62.31 | 76.66 | 73.25 | 78.54 | 64.97 | 72.69 |
| Actinobacteria | 28.59 | 17.05 | 19.93 | 15.93 | 26.75 | 20.38 |
| Bacteroidetes | 3.65 | 1.74 | 2.04 | 1.15 | 2.66 | 2.15 |
| Cyanobacteria/Chloroplast | 2.34 | 1.96 | 1.99 | 1.84 | 2.23 | 2.03 |
| Verrucomicrobia | 1.95 | 1.21 | 1.86 | 1.36 | 1.96 | 1.60 |
| Firmicutes | 0.85 | 1.29 | 0.69 | 0.94 | 1.26 | 0.94 |
| Planctomycetes | 0.25 | 0.09 | 0.20 | 0.13 | 0.15 | 0.17 |
| Gemmatimonadetes | 0.02 | 0.00 | 0.01 | 0.03 | 0.01 | 0.02 |
| Chlorobi | 0.01 | 0.00 | 0.00 | 0.01 | 0.00 | 0.01 |
| Chloroflexi | 0.01 | 0.00 | 0.00 | 0.00 | 0.00 | 0.01 |
| Acidobacteria | 0.00 | 0.00 | 0.00 | 0.02 | 0.01 | 0.00 |
| WS3 | 0.00 | 0.00 | 0.00 | 0.00 | 0.01 | 0.00 |
| Armatimonadetes | 0.00 | 0.01 | 0.00 | 0.00 | 0.00 | 0.00 |
| SR1 | 0.00 | 0.00 | 0.00 | 0.01 | 0.00 | 0.00 |
| unclassified | 0.03 | 0.00 | 0.03 | 0.04 | 0.00 | 0.03 |

**Table S2.** Taxonomic composition of the planktonic bacterial communities in Lake Sayrama (at the genus level)

| Genus | Phylum | Relative Abundance (%) | | | | | |
| --- | --- | --- | --- | --- | --- | --- | --- |
|  |  | Site 1 | Site 3 | Site 4 | Site 5 | Site 6 | Average |
| *Acinetobacter* | Proteobacteria | 53.33 | 55.49 | 63.58 | 70.02 | 53.23 | 59.13 |
| *Ilumatobacter* | Proteobacteria | 15.85 | 12.28 | 12.12 | 10.74 | 17.63 | 13.72 |
| *Loktanella* | Proteobacteria | 0.80 | 4.57 | 1.43 | 1.48 | 3.17 | 2.29 |
| *GpIIa* | Cyanobacteria/Chloroplast | 2.34 | 1.96 | 1.99 | 1.84 | 2.22 | 2.07 |
| *Cryobacterium* | Actinobacteria | 3.46 | 1.01 | 2.04 | 1.37 | 1.69 | 1.91 |
| *Agrococcus* | Actinobacteria | 3.15 | 0.92 | 1.61 | 0.86 | 1.69 | 1.65 |
| *Ornithinibacter* | Actinobacteria | 1.49 | 1.13 | 1.31 | 0.72 | 1.86 | 1.30 |
| *Spartobacteria_genera_incertae_sedis* | Verrucomicrobia | 1.51 | 0.90 | 1.29 | 1.09 | 1.52 | 1.26 |
| *Pseudomonas* | Proteobacteria | 0.35 | 3.08 | 0.26 | 1.34 | 0.19 | 1.04 |
| *Algoriphagus* | Bacteroidetes | 0.94 | 1.42 | 0.75 | 0.48 | 1.42 | 1.00 |
| *Psychrobacter* | Proteobacteria | 0.25 | 0.91 | 0.08 | 1.47 | 0.36 | 0.61 |
| *Limnohabitans* | Proteobacteria | 0.70 | 0.58 | 0.38 | 0.15 | 0.39 | 0.44 |
| *Planococcus* | Firmicutes | 0.19 | 0.92 | 0.24 | 0.33 | 0.31 | 0.40 |
| *Porphyrobacter* | Proteobacteria | 0.17 | 0.72 | 0.34 | 0.11 | 0.29 | 0.33 |
| *Rheinheimera* | Proteobacteria | 0.00 | 1.28 | 0.06 | 0.21 | 0.01 | 0.31 |
| *Luteolibacter* | Verrucomicrobia | 0.32 | 0.24 | 0.48 | 0.21 | 0.28 | 0.31 |
| *Brumimicrobium* | Bacteroidetes | 0.41 | 0.00 | 0.56 | 0.13 | 0.12 | 0.24 |
| *Methylophilus* | Proteobacteria | 0.24 | 0.34 | 0.22 | 0.12 | 0.28 | 0.24 |
| *Pasteuria* | Firmicutes | 0.40 | 0.14 | 0.31 | 0.13 | 0.21 | 0.24 |
| *Kerstersia* | Proteobacteria | 0.16 | 0.38 | 0.20 | 0.12 | 0.15 | 0.20 |
| *Gracilimonas* | Bacteroidetes | 0.30 | 0.09 | 0.16 | 0.05 | 0.23 | 0.17 |
| *Blastopirellula* | Planctomycetes | 0.25 | 0.08 | 0.19 | 0.11 | 0.15 | 0.16 |
| *Exiguobacterium* | Firmicutes | 0.05 | 0.03 | 0.01 | 0.12 | 0.50 | 0.14 |
| Others |  | 2.01 | 1.18 | 1.36 | 2.00 | 1.45 | 1.60 |
| unclassified |  | 11.41 | 10.42 | 9.10 | 4.85 | 10.67 | 9.29 |

**Table S3.** Taxonomic composition of the sedimental bacterial communities in Lake Sayram (at the phylum level)

| Phylum | Relative Abundance (%) | | | | | |
| --- | --- | --- | --- | --- | --- | --- |
|  | Site 1 | Site 2 | Site 4 | Site 5 | Site 6 | Average |
| Proteobacteria | 46.16 | 46.72 | 45.99 | 43.02 | 45.10 | 45.40 |
| Actinobacteria | 11.53 | 10.36 | 8.27 | 7.20 | 8.18 | 9.11 |
| Acidobacteria | 4.47 | 6.32 | 4.05 | 5.49 | 4.71 | 5.01 |
| Chlorobi | 10.81 | 0.58 | 4.96 | 2.65 | 4.67 | 4.73 |
| Bacteroidetes | 1.93 | 8.90 | 1.84 | 7.51 | 2.93 | 4.62 |
| Gemmatimonadetes | 2.23 | 2.02 | 5.19 | 4.85 | 4.88 | 3.83 |
| Nitrospira | 2.39 | 2.75 | 3.83 | 3.86 | 3.71 | 3.31 |
| Planctomycetes | 1.91 | 4.76 | 2.56 | 2.60 | 2.60 | 2.89 |
| Firmicutes | 2.00 | 2.94 | 2.80 | 2.63 | 2.86 | 2.65 |
| Verrucomicrobia | 1.77 | 2.99 | 1.20 | 2.14 | 2.11 | 2.04 |
| Chloroflexi | 1.68 | 1.72 | 1.71 | 2.52 | 1.76 | 1.88 |
| WS3 | 0.62 | 1.04 | 2.91 | 1.23 | 2.27 | 1.61 |
| TM7 | 0.32 | 0.58 | 0.76 | 1.80 | 1.32 | 0.96 |
| OD1 | 0.58 | 0.76 | 0.67 | 1.54 | 0.70 | 0.85 |
| Armatimonadetes | 0.17 | 0.55 | 0.98 | 0.46 | 0.45 | 0.52 |
| Chlamydiae | 0.47 | 0.33 | 0.30 | 0.54 | 0.68 | 0.46 |
| Cyanobacteria/Chloroplast | 0.15 | 0.27 | 0.13 | 0.56 | 0.19 | 0.26 |
| Fibrobacteres | 0.22 | 0.09 | 0.21 | 0.20 | 0.27 | 0.20 |
| Elusimicrobia | 0.19 | 0.12 | 0.16 | 0.30 | 0.18 | 0.19 |
| Deinococcus-Thermus | 0.23 | 0.16 | 0.08 | 0.05 | 0.02 | 0.11 |
| Others | 0.11 | 0.12 | 0.12 | 0.23 | 0.08 | 0.13 |
| unclassified | 10.09 | 5.92 | 11.31 | 8.64 | 10.33 | 9.26 |

**Table S4.** Taxonomic composition of the sedimental bacterial communities in Lake Sayrama (at the genus level)

| Genus | Phylum | Relative Abundance (%) | | | | | |
| --- | --- | --- | --- | --- | --- | --- | --- |
|  |  | Site 1 | Site 2 | Site 4 | Site 5 | Site 6 | Average |
| *Ignavibacterium* | Chlorobi | 10.81 | 0.58 | 4.96 | 2.65 | 4.67 | 4.73 |
| *Thioprofundum* | Proteobacteria | 1.03 | 5.84 | 2.52 | 5.49 | 5.49 | 4.07 |
| *Gemmatimonas* | Gemmatimonadetes | 2.17 | 1.99 | 4.73 | 4.53 | 4.48 | 3.58 |
| *Thiobacter* | Proteobacteria | 6.37 | 1.41 | 3.89 | 2.47 | 3.57 | 3.54 |
| *Nitrospira* | Nitrospira | 2.39 | 2.75 | 3.82 | 3.86 | 3.71 | 3.31 |
| *Acinetobacter* | Proteobacteria | 1.08 | 11.12 | 0.30 | 0.86 | 0.13 | 2.70 |
| *Ohtaekwangia* | Bacteroidetes | 0.25 | 4.89 | 0.73 | 3.27 | 1.22 | 2.07 |
| *Gp16* | Acidobacteria | 2.42 | 2.25 | 1.25 | 1.20 | 1.91 | 1.81 |
| *Desulfohalobium* | Proteobacteria | 3.75 | 0.25 | 2.18 | 0.94 | 1.68 | 1.76 |
| *WS3_genera_incertae_sedis* | WS3 | 0.62 | 1.04 | 2.91 | 1.23 | 2.27 | 1.61 |
| *Blastopirellula* | Planctomycetes | 1.28 | 2.69 | 1.02 | 1.01 | 1.19 | 1.44 |
| *Iamia* | Actinobacteria | 1.28 | 0.71 | 1.37 | 1.16 | 1.30 | 1.16 |
| *Methyloversatilis* | Proteobacteria | 1.63 | 0.48 | 0.79 | 1.13 | 1.36 | 1.08 |
| *TM7_genera_incertae_sedis* | TM7 | 0.32 | 0.58 | 0.76 | 1.80 | 1.32 | 0.96 |
| *Ilumatobacter* | Actinobacteria | 0.47 | 1.93 | 0.49 | 1.12 | 0.75 | 0.95 |
| *Subdivision3_genera_incertae_sedis* | Verrucomicrobia | 1.08 | 0.95 | 0.64 | 0.83 | 0.98 | 0.90 |
| *Steroidobacter* | Proteobacteria | 1.96 | 1.29 | 0.26 | 0.35 | 0.43 | 0.86 |
| *OD1_genera_incertae_sedis* | OD1 | 0.58 | 0.76 | 0.67 | 1.54 | 0.70 | 0.85 |
| *Aciditerrimonas* | Actinobacteria | 0.99 | 0.56 | 1.08 | 0.63 | 0.86 | 0.82 |
| Others |  | 18.87 | 29.82 | 18.90 | 29.20 | 20.24 | 23.41 |
| unclassified |  | 40.98 | 28.32 | 46.99 | 35.01 | 41.77 | 38.61 |


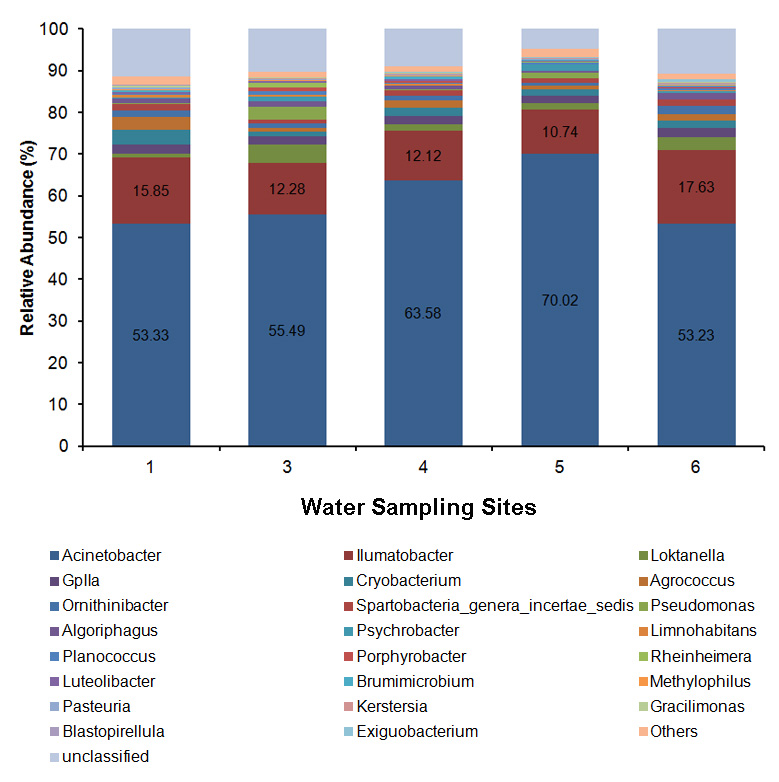


**Figure S1.** The planktonic bacterial diversity of Lake Sayram at the genus level.
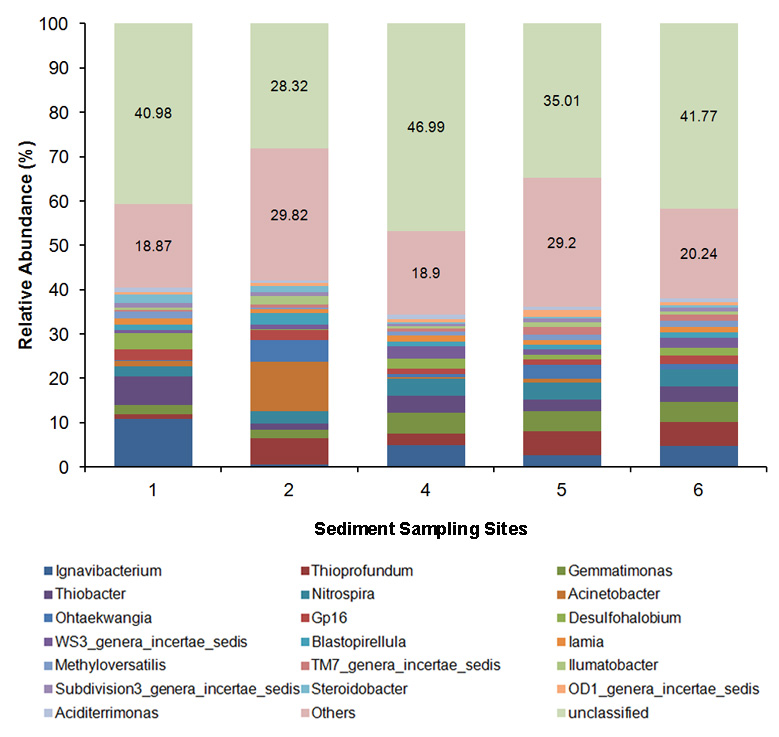


**Figure S2.** Sedimental bacterial diversity of Lake Sayram at the genus level.
